# Characterization and Outcomes of Epileptic Seizures in Mexican Pediatric Patients With Anti-N-Methyl-D-Aspartate Receptor Encephalitis

**DOI:** 10.7759/cureus.8211

**Published:** 2020-05-20

**Authors:** Melissa Chavez-Castillo, Matilde Ruiz-Garcia, Patricia Herrera-Mora

**Affiliations:** 1 Pediatric Neurology, Instituto Nacional de Pediatría, Mexico City, MEX; 2 Pediatric Neurology, Instituto Nacional de Pediatría, Mexico CIty, MEX

**Keywords:** anti-nmdar encephalitis, autoimmune encephalitis, seizures, epilepsy, antiepileptic drugs, outcome, children

## Abstract

Introduction

Anti-N-methyl-D-aspartate receptor (NMDAR) encephalitis is one of the most common autoimmune encephalitides. The frequency of anti-NMDAR encephalitis is known to exceed the frequency of any individual viral encephalitis in young subjects. Epileptic seizures are a cardinal symptom in anti-NMDAR encephalitis; a significant amount of pediatric patients exhibit seizures as the first symptom of the disease, and most of them will develop them during the acute phase. The use of antiepileptic drugs (AEDs) is a cornerstone of the treatment of these patients, but the choice of agent and duration of treatment is currently unknown.

Materials and methods

This was a single-center retrospective review case series of all pediatric patients with a confirmed diagnosis of anti-NMDAR encephalitis and epileptic seizures admitted to the National Institute of Pediatrics in Mexico City from January 2012 to July 2019.

Results

We included a total of 31 patients (males 64.5%, median age: 10 years). No patient showed evidence of teratoma; only 38% of cases had a viral prodrome. Most patients initially exhibited psychiatric symptoms (51%), but the leading cause in soliciting medical assistance was the presence of epileptic seizures (71%). About 85% of patients presented epileptic seizures during the course of the illness, predominantly focal onset seizures (42% focal to bilateral tonic-clonic seizures, 32% focal seizures with impaired awareness). Electroencephalogram (EEG) was abnormal in 97% of patients; the characteristic extreme delta brush pattern was found in 9% of patients. Two AEDs on average were required to control seizures during the acute stage. In six (19%) patients, human herpesvirus (HHV) was detected in cerebrospinal fluid (CSF); all of them had epileptic seizures, which were more resistant to pharmacological treatment during the acute phase, requiring a higher number of AED (median 2.5 vs. 2). The development of epilepsy after acute encephalitis was uncommon; at 24 months, only one patient continued to have epileptic seizures. One of the factors most closely related to the persistence of epileptic seizures was the inadequate response to immunotherapy after four weeks. The functional prognosis was generally good; at a two-year follow-up, only two (10%) patients had a significant disability [modified Rankin Scale (mRS) score: 3-5]; both patients had seizures at a one-year follow-up.

Conclusions

Sustained use of AEDs after the acute phase of anti-NMDAR encephalitis is controversial. We found that the continuation of AEDs after the acute phase could be considered in the following scenarios: status epilepticus (SE), inadequate response to immunotherapy at four weeks, and a high mRS score at discharge and during follow-up. In other cases, discontinuation of AED may be warranted. More studies are needed in our country to replicate these results.

## Introduction

Anti-N-methyl-D-aspartate receptor (NMDAR) encephalitis is one of the most common autoimmune encephalitides and is associated with a characteristic clinical syndrome, which includes the presence of seizures and epilepsy [1]. This disease was first described in 2005 when Vitaliani et al. reported a new syndrome characterized by memory dysfunction, psychiatric symptoms, alterations in consciousness, and autonomic dysfunction in four women with ovarian teratoma [2]. Two years later, specific anti-NMDAR antibodies were found in the cerebrospinal fluid (CSF) of these four patients and eight more with similar neurological symptoms [3]. Its discovery has changed the diagnostic approach to encephalitides in both adults and children and has significantly expanded our knowledge regarding antibody-mediated epilepsy [4].

Women are more commonly affected than men (3:1 ratio), and of all the reported cases of anti-NMDAR encephalitis over a five-year period (September 2007 to February 2011), 65% occurred in patients under the age of 18. Although we lack a specific prevalence study, the frequency of anti-NMDAR encephalitis is known to exceed the frequency of any individual viral encephalitis in young individuals [5].

Epileptic seizures are a cardinal symptom in anti-NMDAR encephalitis; 30% of pediatric patients exhibit them as the first symptom of the disease. Within the first four weeks of disease onset, both children and adults develop a similar clinical syndrome [6]. The pathophysiology of epileptic seizures in anti-NMDAR encephalitis is still unclear, but it is believed to be due to an alteration in the balance of the excitatory and inhibitory mechanisms present in the central nervous system (CNS) [7]. The importance and prevalence of the anti-NMDAR in the developing brain could explain why epileptic seizures are more prevalent in pediatric age [8].

During the acute phase, over 80% of patients will develop seizures [9-11]. Both focal and generalized seizures have been reported. Different studies agree that the most common type of seizure during the acute phase is the generalized tonic-clonic, followed by focal seizures afterward [7,10,11]. Therefore, the use of antiepileptic drugs (AEDs) is a cornerstone in the treatment of these patients, although the choice of agent and duration of AED treatment is currently unknown. The incidence of status epilepticus (SE) in anti-NMDAR encephalitis is approximately 25-50%; about 35% will develop refractory SE [10].

Electroencephalogram (EEG) is abnormal in over 90% of patients. The most common finding is generalized background slowing with a frontotemporal predominance [8,9]. Although most patients develop seizures during disease progression, epileptic activity is a rare finding. It is found in 24-50% of cases, especially during the early stages [12]. The EEG pattern known as extreme delta brush is a unique finding in anti-NMDAR encephalitis. This finding is characterized by a 1-3 Hz rhythmic delta activity with superimposed outbreaks of beta activity at 20-30 Hz. It is transiently found in 30% of adult patients and is associated with a more extended hospitalization [13]. The frequency is lower in pediatric patients (5%), and its association with prognosis is unclear [14,15].

In Mexico, there is a scarcity of studies regarding anti-NMDAR encephalitis, and most of the available information comes from case reports and small case series of adult patients [16,17]. This study aimed to describe the characteristics and outcomes of epileptic seizures, EEG findings, and treatment choices of pediatric patients with anti-NMDAR encephalitis and epileptic seizures at the National Institute of Pediatrics in Mexico City.

## Materials and methods

This was a single-center retrospective study of pediatric patients (<18 years) with a diagnosis of anti-NMDAR encephalitis and epileptic seizures admitted to the National Pediatrics Institute from January 2012 to July 2019. The diagnosis was confirmed by the presence of anti-NMDA receptor antibodies in CSF. We excluded patients with a previous history of epilepsy or with incomplete information in medical records. The local ethics committee approved this protocol.

Patient demographics and the presence of prior infection or tumor were recorded. Epileptic seizures were classified according to the International League Against Epilepsy (ILAE) 2017 guidelines published by Scheffer et al. [18]. Drug-resistant epilepsy was defined as seizures occurring despite two AEDs at appropriate doses. EEG patterns were classified based on the presence of the following features: 1) extreme delta brush; 2) generalized background slowness; 3) focal slowness; 4) epileptic activity; and 5) SE. First and second-line immunomodulatory treatment information was documented. The modified Rankin Scale (mRS) was used during hospitalization, and at 12 and 24 months to quantify the patient's functional status. Finally, the number and type of AEDs were determined at discharge as well as at 12- and 24-month follow-ups.

## Results

Forty-one patients diagnosed with anti-NMDAR encephalitis were admitted to the National Institute of Pediatrics from January 2012 to July 2019, which represented 4.3% of all admissions to the pediatric neurology department at the time. Thirty-five patients (85%) developed seizures during the disease. Four of the patients who had seizures were excluded from the study as one of them had a prior diagnosis of epilepsy, and the other three did not have a complete medical record available for analysis. We eventually chose 31 patients, and 20 (64.5%) among them were male; the median age was 10 years (range: 1-16). The initial clinical and paraclinical characteristics of the patients are listed in Table 1.

**Table 1:** Initial clinical and paraclinical features of the enrolled subjects EEG: electroencephalogram; CSF: cerebrospinal fluid: HHV: human herpesvirus

Characteristics	Values (n=31)
Male gender, n (%)	20 (64.5%)
Age (years), median (range)	10 (1-16)
Associated tumor, n (%)	0 (0%)
Prodromal symptoms, n (%)	12 (38%)
Initial symptom, n (%)	
Psychiatric features	16 (51%)
Epileptic seizures	13 (42%)
Non-epileptic paroxysmal events	2 (7%)
Reason for medical consultation, n (%)	
Epileptic seizures	22 (71%)
Psychiatric features	6 (19%)
Non-epileptic paroxysmal events	3 (9%)
Abnormal EEG, n (%)	29 (97%)
Diffuse slow activity	13 (42%)
Epileptiform activity	11 (35%)
Extreme delta brush pattern	3 (9%)
Focal slow activity	1 (3%)
Continuous epileptiform discharges	1 (3%)
Abnormal CSF analysis, n (%)	17 (55%)
Presence of HHV	6 (19%)

None of the patients was found to have any type of neoplasm during the diagnostic approach or follow-up. Twelve patients (38%) had a history of febrile illness preceding the symptom onset. The initial clinical manifestations in the subjects were as follows: 16 patients (51%) presented with psychiatric symptoms, 13 (42%) with epileptic seizures, and two (7%) had with non-epileptic paroxysmal events. In 22 patients (71%), the reason for consultation was the presence of epileptic seizures, it was psychiatric and behavioral disorders in six (19%), and non-epileptic paroxysmal events in three (9%).

The seizure semiology was as follows: 13 patients (42%) developed focal to bilateral tonic-clonic seizures; 10 (32%) developed focal with impaired awareness; six (19%) had generalized tonic-clonic seizures. In two patients (6%), seizure onset was unknown. Sixteen patients (52%) developed SE during the course of the illness; 11 (35%) had non-refractory SE, four (13%) refractory SE, and one patient (3%) had super-refractory SE (Table 2).

**Table 2:** Seizure characteristics, treatment, and outcomes AED: antiepileptic drug; SE: status epilepticus

Characteristics	Values (n=31)
Seizure onset, n (%)	
Focal to bilateral tonic-clonic	13 (42%)
Focal with impaired awareness	10 (32%)
Generalized tonic-clonic	6 (19%)
Unkown onset	2 (7%)
Presence of SE, n (%)	16 (52%)
Established SE	11 (35%)
Refractory SE	4 (13%)
Super-refractory SE	1 (3%)
Number of AED used, median (range)	2 (1-5)
AED treatment at discharge, n (%)	29 (94%)
AED treatment at 1-year follow-up, n (%)	26 (89%)
AED treatment at 2-year follow-up, n (%)	11 (55%)
Seizures at 1-year follow-up, n (%)	4 (13%)
Seizures at 2-year follow-up, n (%)	1 (3%)

EEG was performed during the acute phase in 30 patients (97%), and 29 (97%) had an abnormal EEG. Extreme delta brush was present in three (9%), diffuse slow activity in 13 (42%), and epileptiform activity was detected in 11 patients (35%). One patient (3%) presented focal slow activity, and continuous epileptiform discharges were found in one patient (3%). AED treatment was given to 30 patients (97%) during acute disease. The median number of AEDs given was two, with a minimum of one AED and a maximum of five. Five patients (16%) were classified as having drug-resistant epilepsy during the acute period of the disease. A total of 15 patients (48%) were on valproic acid (VPA), making it the most frequently used AED. The most common AED combination was VPA and levetiracetam (LEV), which was used in 18 patients (58%). Phenytoin (PTH) was used in 13 patients (42%), and oxcarbazepine was used in seven patients (23%). Other AEDs used included carbamazepine, clonazepam, lacosamide, lamotrigine, and topiramate.

All patients had a CSF analysis, which was reported as abnormal (due to pleocytosis and monocytosis) in 17 patients (55%). In six patients (19%), HHV virus was detected in CSF using polymerase chain reaction (PCR). In five of these patients (83%), human herpesvirus type 7 (HHV-7) was detected, and human herpesvirus type 6 (HHV-6) was detected in one (17%). In 50% of these patients, a prodromal febrile illness was identified as the initial manifestation of the disease. All patients who had herpetic infection had seizures during the course of the disease, and the median number of AED required by these patients was 2.5.

Thirty patients (97%) required hospitalization. The median number of days of hospitalization was 48, with a minimum of 14 days and a maximum of 124 days. Twenty-six patients (84%) received first-line treatment with methylprednisolone and intravenous immunoglobulin G (IVIG). In five patients (16%), the first-line treatment was IVIG. Fifteen patients (48%) required second-line treatment: 14 of them (93%) received rituximab, and one (3%) received azathioprine. A significant clinical improvement was seen in 19 patients (61%) four weeks after the onset of immunotherapy; none of these patients presented epileptic seizures during follow-up. Out of the 12 patients (39%) with an inadequate clinical response to immunotherapy, five (41%) persisted with epileptic seizures at the six-month follow-up.

Out of the total sample, 29 patients (94%) were available for follow-up in the year after diagnosis; 20 patients (64%) had a follow-up for two years. Four patients (13%) persisted with epileptic seizures after a year; only one patient (3%) continued to exhibit epileptic seizures at the two-year follow-up. Of the five patients classified with drug-resistant epilepsy during the acute illness, only one continued with the same diagnosis after a two-year follow-up. At discharge, 29 patients (94%) continued with an AED. At a one-year follow-up, 26 out of 29 patients (89%) still had an AED. Out of the 20 patients with two years of follow-up, 11 (55%) continued with AED treatment. Twenty-one patients (72%) had a new EEG at the one-year follow-up. Findings were as follows: eight patients (38%) had generalized slowing; seven patients (33%) had a normal EEG; five patients (24%) had epileptiform activity; focal slowing was identified in one patient (5%).

**Figure 1:**
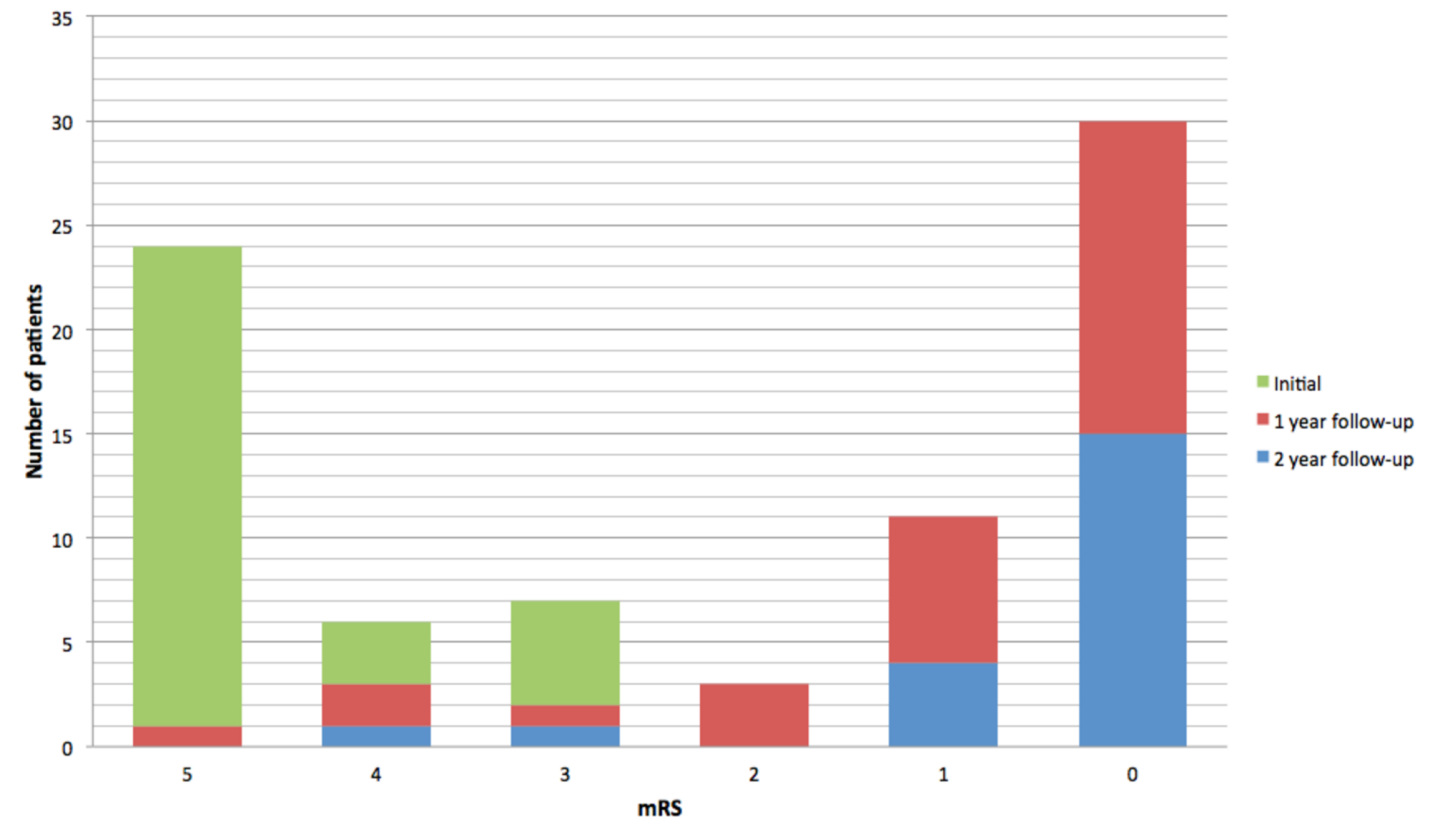
Functional outcome as measured by mRS at admission and follow-up mRS: modified Rankin Scale

During the acute illness, 23 patients (74%) had a maximal mRS score of 5, three patients (10%) had an mRS score of 4, and five patients (16%) had an mRS score of 3. All patients had an improvement in the mRS score at one- and two-year follow-up after initial diagnosis. At one-year follow-up, 15 out of 29 patients (52%) had an mRS score of 0; seven (24%) patients had an mRS score of 1; three patients (10%) had an mRS score of 2, and four (14%) patients had an unfavorable outcome (mRS score: 3-5). At the two-year follow-up, only two patients (10%) presented an unfavorable outcome (Figure 1).

## Discussion

Our study showed a predominance of affected males, which was in contrast with previous reports [5]. To our knowledge, this demographic profile has not been previously highlighted and could be relevant in the characterization of the disease in the pediatric population.

The presence of teratoma was intentionally sought, both in the acute phase and at follow-up, but no cases were found. The paraneoplastic form of anti-NMDAR encephalitis is known to be less common in the pediatric population [2,8,9]. Only 38% of cases had a viral prodrome, and this supports the theory that there are other mechanisms not yet well studied that lead to the development of anti-NMDAR encephalitis in pediatric patients apart from paraneoplastic and post-infectious etiologies. HHV-6 and HHV-7 could play an essential role in this alternative hypothesis.

Most patients started their illness with psychiatric symptoms, but the leading cause in soliciting medical assistance was the presence of epileptic seizures. This finding suggests that psychiatric disorders are the most common initial symptom in both children and adults but can be minimized in the pediatric population. In this series, 85% of patients had epileptic seizures, which was higher than what was previously reported (60-80%) [8,10,11]. Although there is no specific type of epileptic seizure for this entity, focal seizures were predominantly found in this study.

The mean number of AEDs required for the control of seizures during the acute stage was two, which is consistent with previous studies [10,11]. The development of epilepsy after acute encephalitis was uncommon; at 12 months, four patients persisted with seizures, and all of them presented SE during the acute illness. At 24 months, only one patient continued to have seizures. One of the factors most closely related to the persistence of post-acute encephalitis seizures was a poor response to immunomodulatory treatment at four weeks.

There is very little information available about the relationship between the presence of HHV in CSF and anti-NMDAR encephalitis [19]. In this series, all patients with HHV infection had epileptic seizures, which were more resistant to pharmacological treatment during the acute phase, requiring a higher number of AED on average. Only 50% had a history of fever, and none of them had a characteristic picture of herpetic encephalitis. Only one of these patients persisted with seizures at 12 months, and none of them had seizures at 24 months. This finding suggests that despite having more significant morbidity during the acute phase, patients with HHV-6 and HHV-7 infection do not necessarily have a higher risk of epilepsy in the long term. The characteristic extreme delta brush EEG pattern was found in 9% of patients; this pattern was found in a lower percentage than in adult cohorts (30%). However, it was more prevalent compared to results in other pediatric series (5%). A notable fact is that this series had a higher number of pediatric patients included compared to previously published results [14,15]. None of the patients with extreme delta brush EEG pattern persisted with long-term epilepsy.

As previously reported, the functional prognosis of these patients was generally good. At the two-year follow-up, only two patients (10%) had a significant disability (mRS: 3-5); both patients had seizures at the one-year follow-up. The median age of these patients was 3.5 years, which suggests that lower age is a risk factor for a poor functional outcome, which is consistent with previously published data [20].

Very few studies have focussed on the semiology of epileptic seizures and the transition to epilepsy in patients with anti-NMDAR encephalitis so far, and none of them are specific to the pediatric population. To our knowledge, this is the first study describing the clinical manifestations of seizures in pediatric patients with anti-NMDAR encephalitis. Sustained use of AEDs after the acute phase of anti-NMDAR encephalitis is controversial. The low prevalence of long-term epilepsy, as well as the absence of seizures in patients with an adequate response to immunomodulatory treatment, support the theory that seizures present in patients with anti-NMDAR encephalitis could be classified as symptomatic, and these patients do not have to carry the burden of a lifelong diagnosis of epilepsy.

## Conclusions

Based on our findings, we propose that the continuation of AEDs after the acute phase could be considered in the following scenarios: SE, inadequate response to immunotherapy at four weeks, and a high mRS score at discharge and during follow-up. In all other cases, discontinuation of AED may be warranted. More studies are needed in our country to replicate these results.

## References

[1] Dalmau J, Lancaster E, Martinez-Hernandez E, Rosenfeld MR, Balice-Gordon R (2011). Clinical experience and laboratory investigations in patients with anti-NMDAR encephalitis. Lancet Neurol.

[2] Vitaliani R, Mason W, Ances B, Zwerdling T, Jiang Z, Dalmau J (2005). Paraneoplastic encephalitis, psychiatric symptoms, and hypoventilation in ovarian teratoma. Ann Neurol.

[3] Dalmau J, Tüzün E, Wu HY (2007). Paraneoplastic anti-N-methyl-D-aspartate receptor encephalitis associated with ovarian teratoma. Ann Neurol.

[4] Bien C, Holtkamp M (2017). "Autoimmune epilepsy”: encephalitis with autoantibodies for epileptologists. Epilepsy Curr.

[5] Gable MS, Sheriff H, Dalmau J, Tilley DH, Glaser CA (2012). The frequency of autoimmune N-methyl-D-aspartate receptor encephalitis surpasses that of individual viral etiologies in young individuals enrolled in the California Encephalitis Project. Clin Infect Dis.

[6] Titulaer MJ, McCracken L, Gabilondo I (2013). Treatment and prognostic factors for long-term outcome in patients with anti-NMDA receptor encephalitis: an observational cohort study. Lancet Neurol.

[7] Cooray GK, Sengupta B, Douglas P, Englund M, Wickstrom R, Friston K (2015). Characterising seizures in anti-NMDA-receptor encephalitis with dynamic causal modelling. Neuroimage.

[8] Florance NR, Davis RL, Lam C (2009). Anti-N-methyl-D-aspartate receptor (NMDAR) encephalitis in children and adolescents. Ann Neurol.

[9] Dalmau J, Gleichman AJ, Hughes EG (2008). Anti-NMDA-receptor encephalitis: case series and analysis of the effects of antibodies. Lancet Neurol.

[10] de Bruijn MAAM, van Sonderen A, van Coevorden-Hameete MH (2019). Evaluation of seizure treatment in anti-LGI1, anti-NMDAR, and anti-GABABR encephalitis. Neurology.

[11] Liu X, Yan B, Wang R, Li C, Chen C, Zhou D, Hong Z (2017). Seizure outcomes in patients with anti-NMDAR encephalitis: a follow-up study. Epilepsia.

[12] Irani SR, Bera K, Waters P (2010). N-methyl-d-aspartate antibody encephalitis: temporal progression of clinical and paraclinical observations in a predominantly non-paraneoplastic disorder of both sexes. Brain.

[13] Schmitt SE, Pargeon K, Frechette ES, Hirsch LJ, Dalmau J, Friedman D (2012). Extreme delta brush: a unique EEG pattern in adults with anti-NMDA receptor encephalitis. Neurology.

[14] Armangue T, Titulaer MJ, Málaga I, Bataller L, Gabilondo I, Graus F, Dalmau J (2013). Pediatric anti-N-methyl-D-aspartate receptor encephalitis-clinical analysis and novel findings in a series of 20 patients. J Pediatr.

[15] Jeannin-Mayer S, André-Obadia N, Rosenberg S, Boutet C, Honnorat J, Antoine JC, Mazzola L (2019). EEG analysis in anti-NMDA receptor encephalitis: description of typical patterns. Clin Neurophysiol.

[16] González-Latapi P, Rodríguez-Violante M, Cervantes-Arriaga A, Calleja-Castillo JM, González-Aguilar A (2014). Encefalitis por anticuerpos antirreceptor de N-metil-D-aspartato (anti-NMDAR): reporte de un caso. (Article in Spanish). Gac Med Mex.

[17] Jiménez-Ruiz A, Cárdenas-Sáenz O, Ruiz-Sandoval JL (2017). Encefalitis autoinmunitaria secundaria a teratoma ovárico: un nuevo síndrome neuropsiquiátrico. Reporte de caso. (Article in Spanish). Ginecol Obstet Mex.

[18] Scheffer IE, Berkovic S, Capovilla G (2017). ILAE classification of the epilepsies: position paper of the ILAE Commission for Classification and Terminology. Epilepsia.

[19] Armangue T, Spatola M, Vlagea A (2018). Frequency, symptoms, risk factors, and outcomes of autoimmune encephalitis after herpes simplex encephalitis: a prospective observational study and retrospective analysis. Lancet Neurol.

[20] Zekeridou A, Karantoni E, Viaccoz A (2015). Treatment and outcome of children and adolescents with N-methyl-D-aspartate receptor encephalitis. J Neurol.

